# Prospective comparison of capillary and venous brain biomarker S100B: capillary samples have large inter-sample variation and poor correlation with venous samples

**DOI:** 10.1186/s12245-019-0239-6

**Published:** 2019-09-02

**Authors:** Tomas Vedin, Mathias Karlsson, Marcus Edelhamre, Mikael Bergenheim, Per-Anders Larsson

**Affiliations:** 1 Department of Clinical Sciences, Lund University, Svartbrödragränden 3-5, 251 87 Helsingborg, Sweden; 2 Department of Clinical Chemistry and Center for Clinical Research, Centralsjukhuset, Karlstad, Sweden; 3 Karlstad Central Hospital, Rosenborgsgatan 9, 652 30 Karlstad, Sweden

**Keywords:** Capillary, Venous, Blood specimen collection, Brain injuries traumatic, S100 calcium-binding protein beta subunit, S100B

## Abstract

**Background:**

Guidelines for the emergency management of mild traumatic brain injury have been used for over a decade and are considered safe. However, they recommend computerized tomography for at least half of these patients. The Scandinavian Neurotrauma Committee guideline uses serum S100B protein level to rule out intracranial hemorrhage. Analysis of capillary serum S100B protein level has not yet been employed for this purpose. The primary aim of this study was to investigate the correlation and agreement of capillary and venous serum S100B protein level over a spectrum of concentrations typical for mild traumatic brain injury.

**Methods:**

Eighteen patients with traumatic intracranial hemorrhage and 39 volunteers without trauma to the head within the past 7 days were recruited. Blood was sampled from patients with intracranial hemorrhage daily up to four consecutive days and healthy volunteers were sampled once during the study. One venous and two capillary samples were drawn at each sampling event. Samples were analyzed using the Cobas e411 S100 electrochemiluminescence assay.

**Results:**

Median serum S100B protein level of capillary sampling 1 was 0.12 (IQR 0.075–0.21) μg/l and median serum S100B protein level of capillary sampling 2 was 0.13 (IQR 0.08–0.22) μg/l. Median serum S100B protein level of all venous samples was 0.05 (IQR 0.03–0.07) μg/l.

Correlation plots of capillary and venous samples showed poor correlation and Bland-Altman plots showed a large dispersion of samples and wide limits of agreement.

**Conclusion:**

The results of this study indicate that correlation and agreement between capillary and venous samples are low, and because of this, we cannot recommend studies on capillary serum S100B protein level to rule out intracranial hemorrhage in mild traumatic brain injury. Given the limitations of the current sampling and analysis methods of capillary protein S100B protein level, we conclude that evaluating its predictive ability to rule out intracranial hemorrhage should be withheld until more reliable methods can be incorporated into the study design.

**Electronic supplementary material:**

The online version of this article (10.1186/s12245-019-0239-6) contains supplementary material, which is available to authorized users.

## Background

Guidelines for the emergency management of adult mild traumatic brain injury (mTBI) have been used for more than a decade and adhering to them might improve clinical practice [1, 2]. These guidelines recommend computerized tomography (CT) head scans for half of mTBI patients [2]. The incidence of intracranial hemorrhage (IH) in a general emergency department cohort of patients with TBI ranges from 4 to 8%. This yields 6–12 CTs to find one IH [2, 3].

Protein S100B, a brain damage biomarker, is secreted by astrocytes. Secondary sources include adipocytes, chondrocytes, malignant melanoma cells, Schwann cells, and leukocytes [4, 5]. Protein S100B regulates calcium homeostasis and has neurotrophic effects in the nanomolar range and neurotoxic effects in the micromolar range [6, 7]. It passes from cerebrospinal fluid to blood and is eliminated by the kidneys. The half-life is 25–97 min [8, 9]. Significant amounts of protein S100B in leukocytes has been reported, and it is possible that swift analysis of S100B samples would help avoid false high results because of leukocyte lysis [4].

The latest guideline for adult patients from the Scandinavian Neurotrauma Committee incorporates serum S100B protein level into the clinical decision-making process. The guideline has been validated, and in this study of a North American emergency department cohort with mTBI, it gave a head-CT reduction of 32% [10]. An S100B of < 0.1 μg/l measured within 6 h of trauma has a high negative predictive value and serves as the clinical cutoff for ruling out IH [3]. Two studies have explored capillary sampled blood for serum S100B protein level assay, but no studies have tested if capillary sampled serum S100B protein can be used to rule out IH [11, 12]. Furthermore, the analytical bias has not been extensively explored. The range of serum S100B protein level in mTBI is wide, and these patients might have serum S100B protein levels both below and well above the clinical cutoff of 0.1 μg/l. Even levels up to 1.5 μg/l have been reported [13–17].

Capillary sampling has the advantages of possible point-of-care analysis and no need for venipuncture. This might, for instance, facilitate serum S100B protein level analysis in pediatric patients. However, research on capillary sampling for other biomarkers indicates that it may be unreliable [18–20]. Åstrand et al. concluded that capillary and venous serum S100B protein level assays should be regarded as separate variables but suggested a linear “conversion equation” to predict venous serum S100B protein levels from capillary serum S100B protein levels [10].

## Methods

The primary aim of the study was to investigate the correlation and agreement of capillary and venous serum S100B protein level over a spectrum of concentrations typical for mild traumatic brain injury. The secondary aim was to evaluate the conversion equation’s ability to predict venous values of serum S100B protein level.

The study was conducted as a prospective cohort study at the Department of Neurosurgery, Skåne University Hospital, Lund, where patients with IH and moderate to severe traumatic brain injury (TBI) (Glasgow Coma Scale 3–13) were included and the Helsingborg General Hospital where patients with IH and mild TBI were included (Glasgow Coma Scale 14–15) as defined by the SNC [3]. The study was conducted over 12 months in 2014. A total of 18 patients with IH were included. These patients were recruited by monitoring admissions at the wards and approaching the patients eligible for the study with an invitation to join. Furthermore, 39 healthy volunteers were included. They were recruited by advertising the study among staff and medical students at the hospital.

Inclusion criteria for patients with traumatic brain injury were age ≥ 18 years and traumatic intracranial hemorrhage visible on CT scan. Exclusion criteria were multitrauma, metastasized malignant melanoma, kidney failure, and previous upper extremity amputation. The CT scans were interpreted by the radiologist on-call and reviewed the next day by a senior radiologist.

The inclusion criterion for healthy volunteers was age ≥ 18 years. Exclusion criteria were trauma to the head within the past 7 days, metastasized malignant melanoma, kidney failure, and previous upper extremity amputation.

The rationale for including both healthy volunteers and patients with mild, moderate, and severe degrees of TBI was to make it easier to find samples with relevant concentrations of S100B establish the correlation and agreement over the entire concentration spectrum of serum S100B protein level that is commonly found in patients with mTBI.

This method differs from how a clinical study of prediction would be designed but is praxis in the field of clinical chemistry when studying analytical bias. We did not actively seek to include patients with very high values of S100B as this was not relevant to the aim of this study.

The following sampling algorithm was used:

1. One venous sample and two capillary samples were drawn from the same patient at each sampling event.
2. A limited number of operators (one research fellow and two research nurses) sampled the blood with efforts made not to squeeze the fingertip while sampling.
3. All samples were analyzed on the day of sampling and the same procedures were used for transport and assay.

Blood samples were collected in accordance with the sampling algorithm from in-hospital patients daily until the patient was discharged but for a maximum of four consecutive days. Each sampling event was treated as an independent sampling event and it was therefore not reported from which patient and on what day the samples were taken. Volunteers were sampled once during the study.

Venous blood (4 ml) was sampled from the antecubital vein and placed in regular serum separating tube vacutainers. Capillary blood (400 μL) was sampled from a lancet stab of 2-mm depth in the lateral or medial side of two fingers and placed in two open serum separating tube capillary containers. The serum separating tubes contained a serum separating gel without additives. To extract serum, samples were centrifuged at 2200*g* for 10 min and immediate analysis was performed. No samples were frozen or refrigerated. Samples were analyzed using the Cobas e411 S100 electrochemiluminescence assay (Roche Diagnostics), and the hospitals’ laboratories (accredited by the Swedish Board for Accreditation and Conformity Assessment) performed the analyses. Samples were rejected only in the case of inadequate sample volume and never because of blood clots or hemolysis. The assay had an 18-min processing time, a lower detection limit of 0.005 μg/l, a within-assay coefficient of variability at 1.8%, and a total imprecision of 3.1% for concentrations between 0.08 and 2.13 μg/l [21]. The results were automatically uploaded into the electronic medical records database.

The following “prediction equation” to calculate venous serum S100B protein levels from capillary serum S100B protein levels was put forth by Åstrand et al. [12]:

$$ \mathrm{Venous}\ \mathrm{S}100\mathrm{B}\frac{\upmu \mathrm{g}}{\mathrm{l}}=0.984\times \mathrm{Capillary}\ \mathrm{S}100\mathrm{B}\frac{\upmu \mathrm{g}}{\mathrm{l}}-0.061 $$

The mean prediction error of the equation was 0.064 μg/l, and it could theoretically be used to predict if samples are above or below the clinical cutoff. This equation will be referred to as the “prediction equation” in the current study.

### Statistics

Data were analyzed using the IBM™ Statistical Package for the Social Sciences (SPSS) v. 25 for Mac. Histograms and Shapiro-Wilks formula were used to evaluate normal distribution. Central tendencies were presented as means ± 1.96 standard deviations and as medians with interquartile range (IQR). Samples were only included in statistical analyses if there was a valid venous sample, and two valid capillary samples of serum S100B protein level except for analyses of the prediction equation where only one matching capillary sample was required for inclusion. Numeric differences between sampling methods were treated as unrelated, non-parametric samples, and the Mann-Whitney *U* test was used to evaluate the statistical significance of differences. The samples were compared with correlation plots and Bland-Altman plots. Intercept, slope, and determination coefficient (*R*^2^) with confidence intervals were obtained using linear regression. Correlation plots were logarithmized to force normality and thus put less emphasis on outliers without removing them. Pearson’s correlation coefficient was used to evaluate the correlation of sampling methods in logarithmized correlation graphs. When comparing capillary samples to venous samples with Bland-Altman plots, only venous samples were used on *y*-axis, because venous sample is gold standard [22]. Statistical significance was set to *p* > 0.05. A power calculation was performed using Sample Power 2.0 for SPSS. It was based on the correlation between 16 randomized venous and corresponding capillary serum S100B protein samplings from the publication by Åstrand et al. [12]. The correlation between these samples was 0.53 and with a power of 95%; it would take 36 matching venous and 36 capillary samples to yield statistically significant results.

## Results

**Fig. 1:**
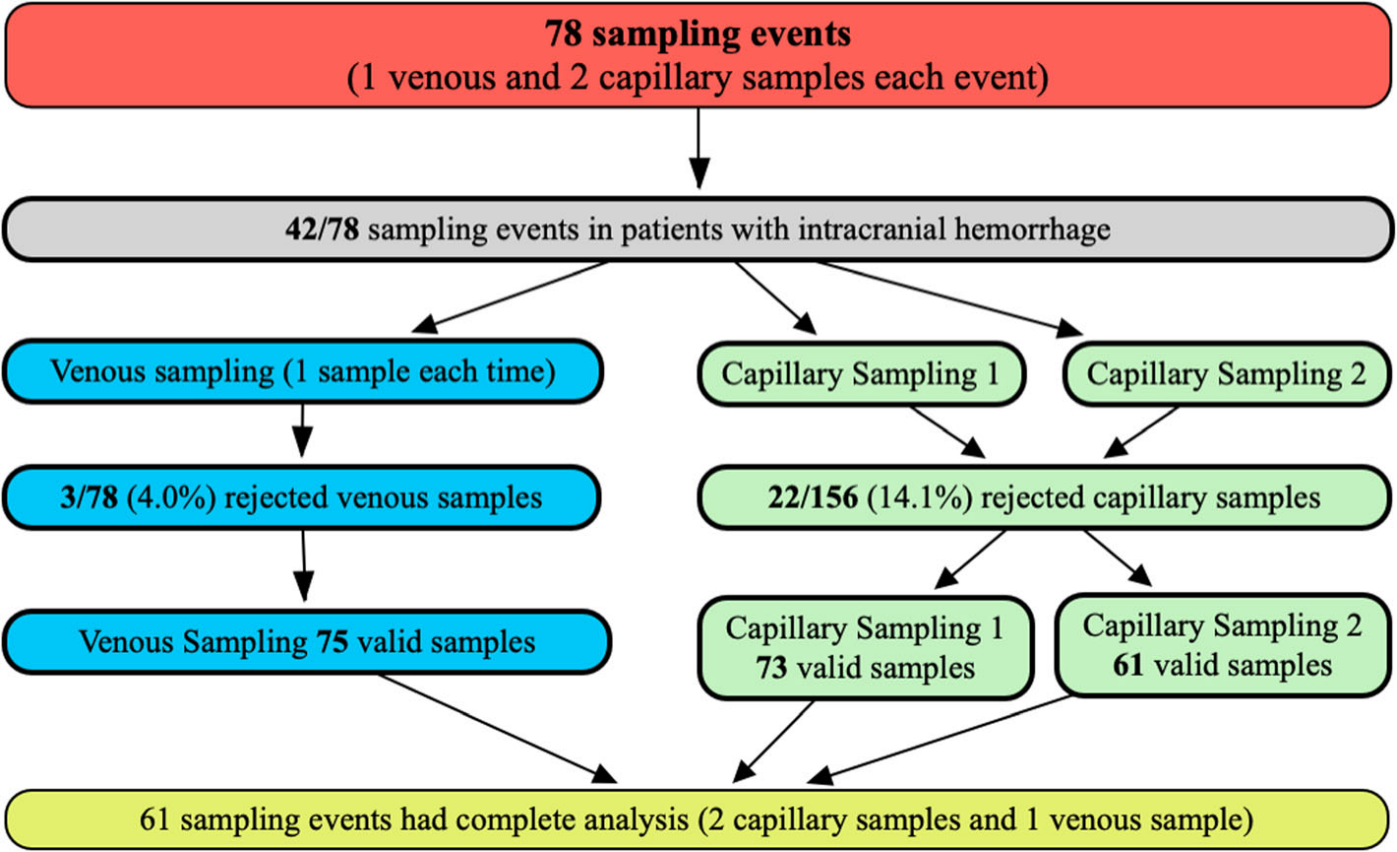
Flow chart of sampling process. Seventy-eight sampling events with one venous and two capillary samples

The 39 healthy volunteers (18 females/21 males) had a median age of 28 (IQR 26–32.75) years and the 18 patients (9 females/9 males) with IH had a median age of 53 (IQR 34.5–70) years. Refer to Fig. 1 for a flow chart of the sampling process. All laboratory rejected samples were rejected because of insufficient sample volume.

Of the 75 sampling events, 42 were from patients with intracranial hemorrhage. A total of 61 samples had corresponding venous and capillary samples. These are the samplings that were presented throughout the present study, except in calculations pertaining to prediction equation.

Of the successfully analyzed venous samples in patients with IH, three patients had four sampling events, one patient had three sampling events, seven patients had two sampling events, and seven patients had one sampling event before discharge (36 samples in total). The three rejected venous samples all came from patients with IH.

**Fig. 2:**
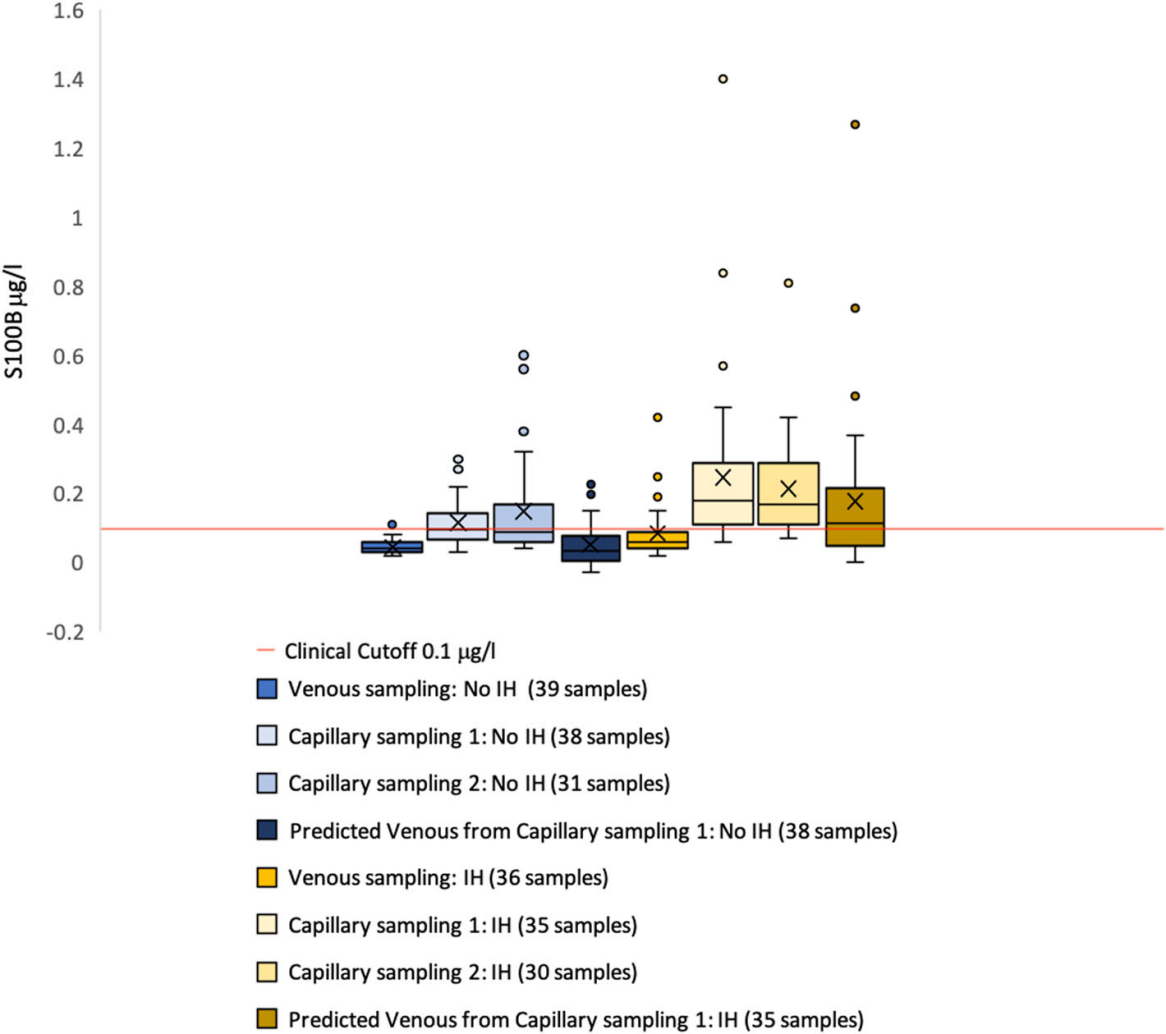
Box-and-whiskers plot of venous and capillary S100B samples. Venous and capillary samplings of serum S100B protein level sampled in patients with and healthy volunteers without intracranial hemorrhage (IH)

The range of venous serum S100B protein level in all patients was 0.03–0.42 μg/l. Refer to Fig. 2 for a box plot of median serum S100B protein level values in patients with and without head trauma and intracranial hemorrhage. Refer to Additional file 1: Table S1 for a complete description of the dispersion capillary and venous S100B levels in patients with intracranial hemorrhage and healthy volunteers without intracranial hemorrhage.

Median serum S100B protein level of all venous samples was 0.05 (IQR 0.03–0.07) μg/l. Median serum S100B protein level of venous samples in healthy volunteers was 0.04 (IQR 0.03–0.06) μg/l. Median serum S100B protein level of venous samples in patients with IH was 0.06 (IQR 0.04–0.09) μg/l. The median difference was 0.0.07 (IQR 0.04–0.15) μg/l between capillary sampling 1 and venous sampling and 0.07 (IQR 0.05–0.17) μg/l between capillary sampling 2 and venous sampling.

Using the Mann-Whitney *U* test, the differences between capillary samples and venous samples in patients with and without intracranial hemorrhage were statistically significant (venous samples no IH versus venous samples IH − *p* = 0.001, capillary sampling 1 no IH versus capillary sampling 1 IH − *p* = 0.002, and capillary sampling 2 no IH versus capillary sampling 2 IH *p* = 0.003). With the same test, the differences between all venous and capillary samples were statistically significant (venous and capillary sampling 1 (*p* < 0.001) and venous and capillary sampling 2 (*p* < 0.001)), but the difference between capillary sampling 1 and capillary sampling 2 was not (*p* = 0.47).

In four of the analyses, venous protein S100B levels were higher than in capillary sample 1. In capillary sample 2, two of these four venous samples were missing paired values, one was equal to the venous sample, and one was less than the venous sample. In the remaining 205 samples, capillary sample values were equal to or higher than venous samples.

**Fig. 3:**
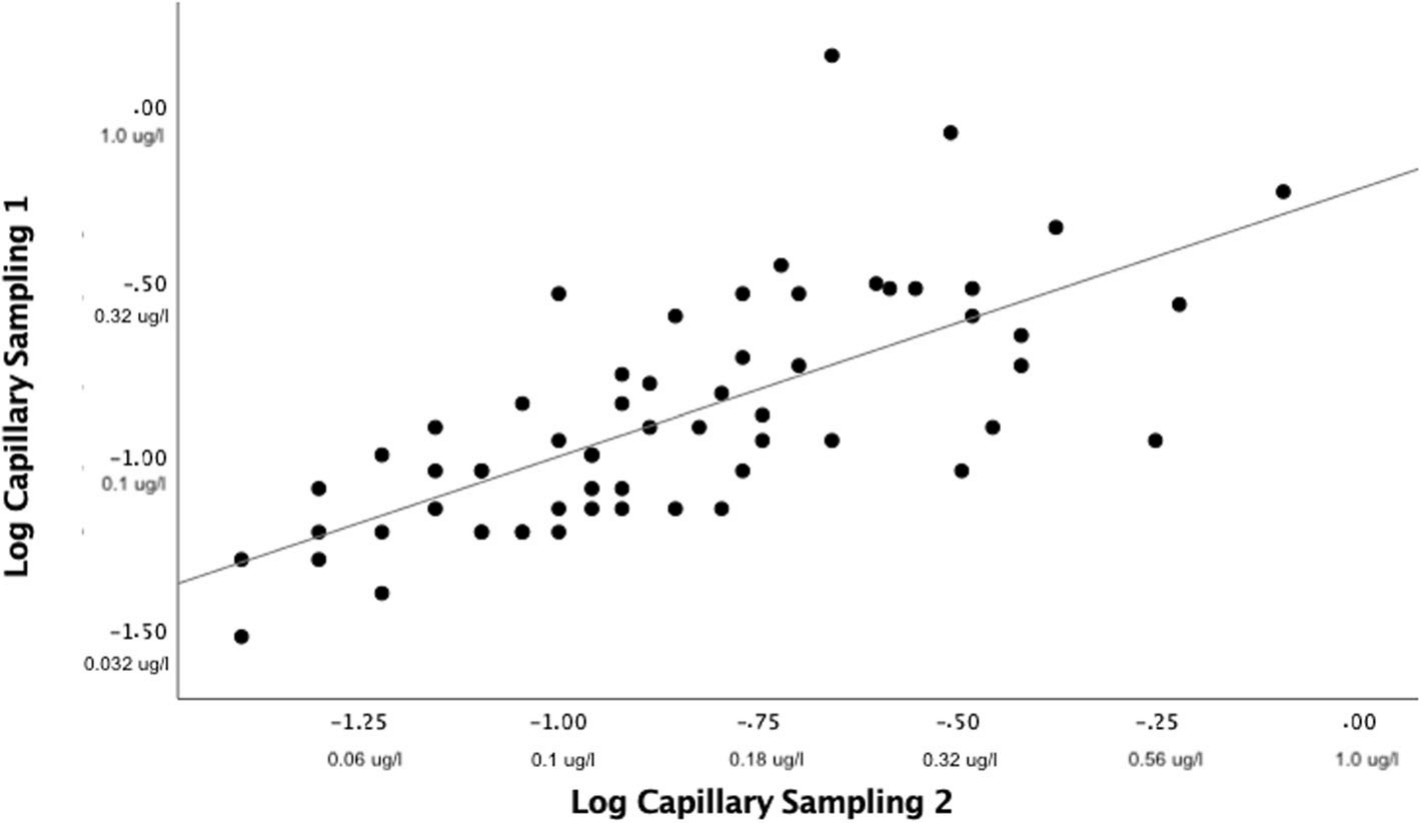
Log correlation plot capillary 1 and capillary 2 S100B. Sixty-one matched samples. Positive numbers with unit μg/l are corresponding non-logarithmized values for illustrational purposes. Pearson’s correlation coefficient = 0.70 (*p* < 0.05). Regression line: intercept = − 0.24 (95% confidence interval − 0.42 to 0.06). Slope 0.77 (95% confidence interval 0.56–0.97). *R*^2^ = 0.49

**Fig. 4:**
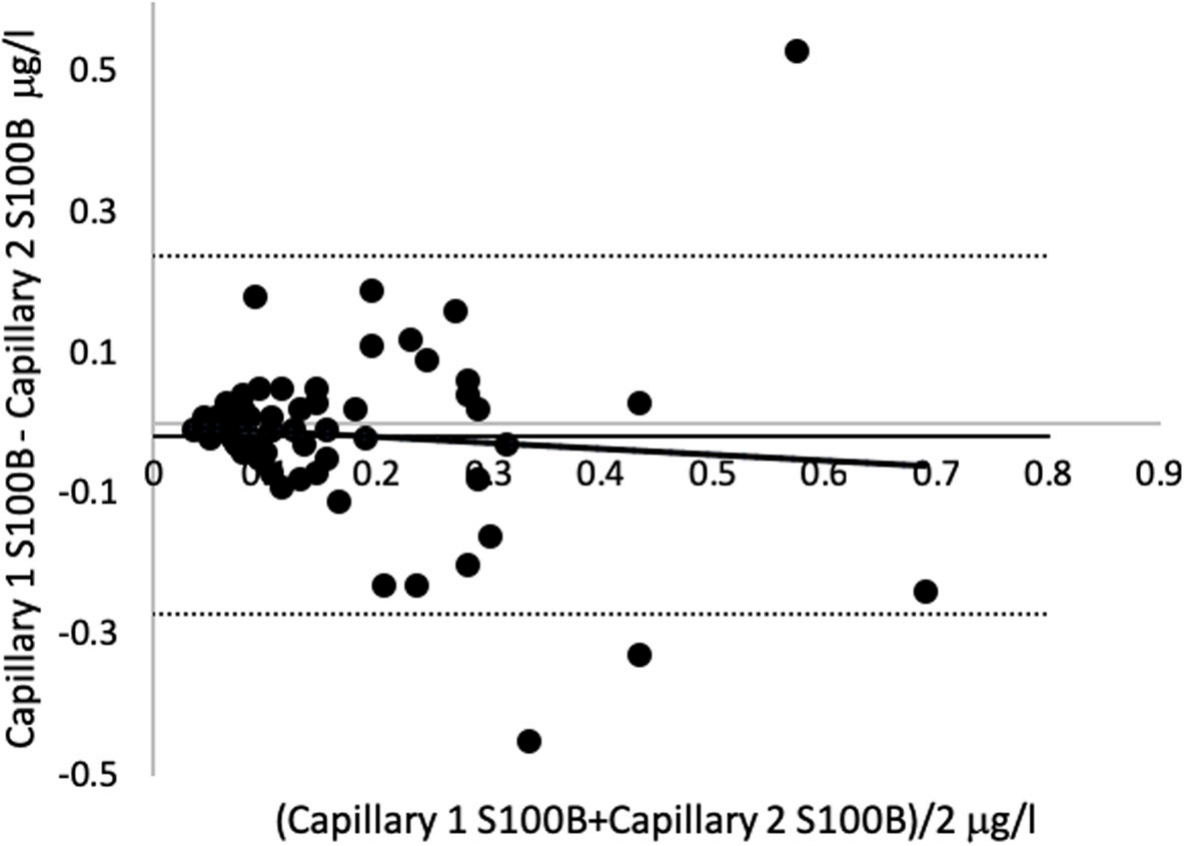
Bland-Altman plot capillary 1 versus capillary 2 S100B. Sixty-one matched samples. Regression line: intercept = − 0.002 (95% CI − 0.06 to 0.05). Slope = − 0.08 (− 0.34–0.18). *R*^2^ = 0.07

A log correlation plot of capillary 1 and capillary 2 showed Pearson’s correlation coefficient < 0.8 (Fig. 3). The line of regression in a Bland-Altman plot of capillary samples showed a slight negative slope and three samples were outside the limits of agreement. Limits of agreement were − 0.27 μg/l and 0.24 μg/l (Fig. 4).

**Fig. 5:**
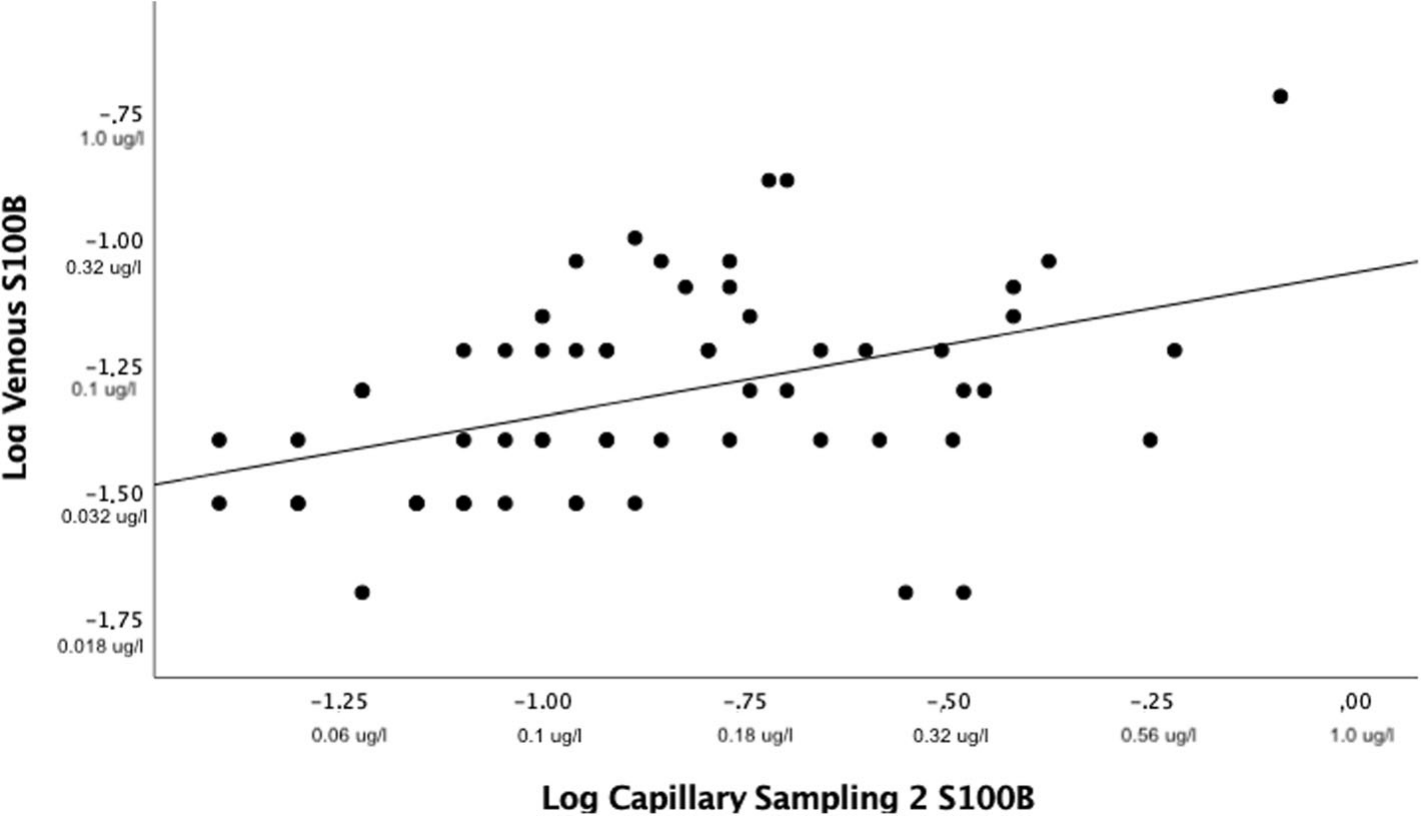
Log correlation plot venous and capillary 2 serum S100B protein level. Sixty-one matched values. Positive numbers with unit μg/l are corresponding non-logarithmized values for illustrational purposes. Pearson’s correlation coefficient of 0.41 (*p* < 0.05). Line of regression: intercept: − 1.07 (95% confidence interval − 1.21 to − 0.92). Slope = 0.28 (95% confidence interval 0.12–0.44)

**Fig. 6:**
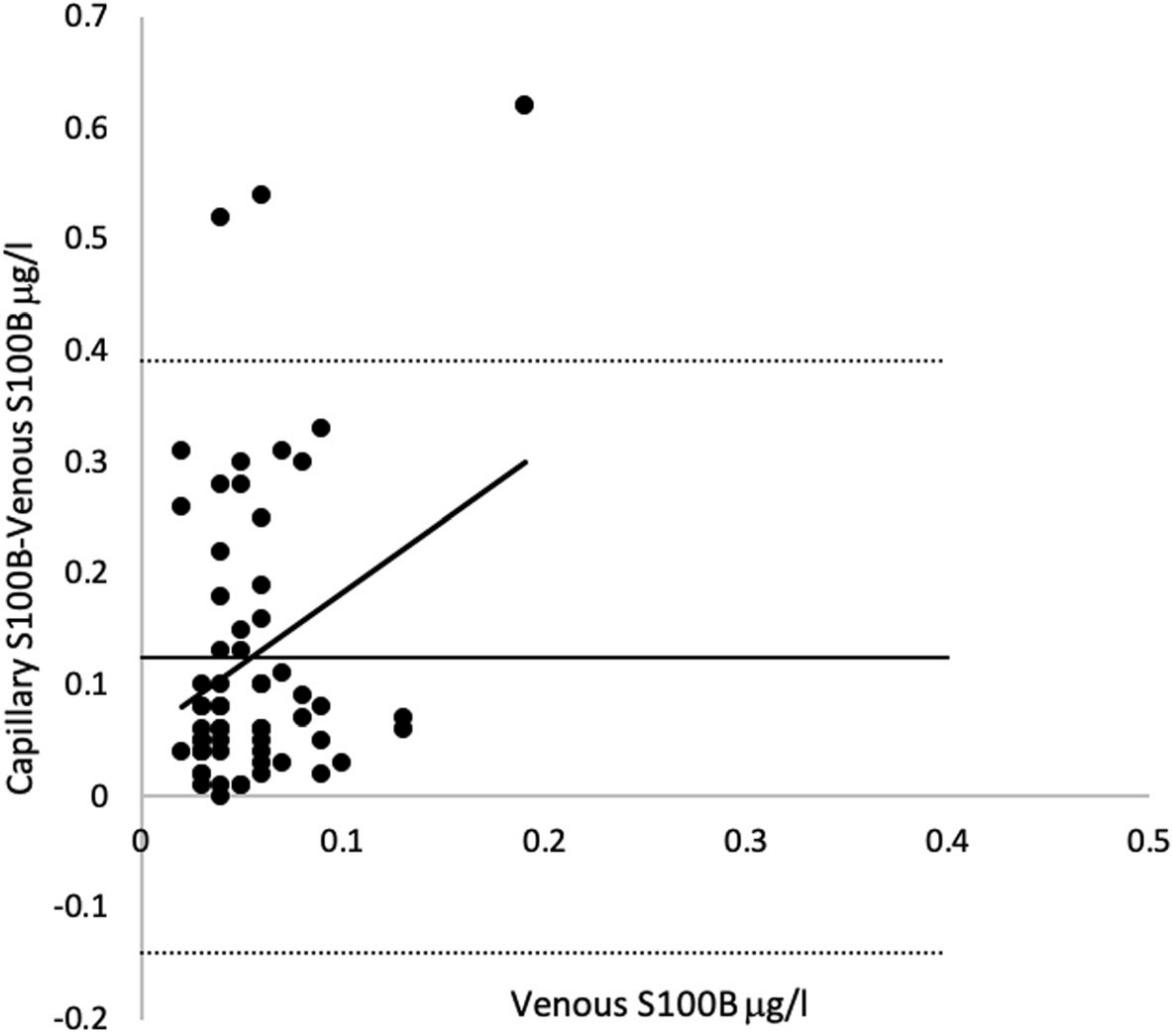
Bland-Altman plot venous versus capillary 2 serum S100B protein level. Sixty-one matched samples. Venous sample used on *y*-axis because the true value is assumed to be venous sample. Regression line: intercept = 0.05 (95% confidence interval 0.04–0.06). Slope 0.06 (95% confidence interval 0.01–0.12). *R*^2^ = 0.08

Log correlation plot of capillary and venous samples showed Pearson’s correlation coefficient < 0.5 (Fig. 5). Bland-Altman plots of capillary samplings 1 and 2 versus venous sampling showed a large dispersion of samples and a steep positive slope of the line of regression. Three samples were outside the limits of agreement in both capillary samplings 1 and 2. Limits of agreement for capillary sampling 1 and venous sampling were − 0.08 μg/l and 0.27 μg/l. Limits of agreement for capillary sampling 2 and venous sampling were − 0.14 μg/l and 0.39 μg/l (Fig. 6. Correlation plots and Bland-Altman plots for capillary sampling 1 versus venous sampling were not included because they were similar to the corresponding plots for capillary sampling 2.

The correlation coefficient was not calculated in predicted venous and venous samples because the predicted venous samples had negative values in some instances.

Prediction equation classified 22/73 (30.1%) samples incorrectly over or under the clinical cutoff in capillary sampling 1 with a mean prediction error of 0.053 (± 0.353) μg/l. In capillary sampling 2, 22/61 (36.1%) samples were incorrectly predicted over or under the clinical cutoff of 0.1 μg/l with a mean prediction error of 0.058 (± 0.251) μg/l. In capillary sampling 1, eight corresponding venous samples were above the clinical cutoff and 2/8 (25%) were predicted below the cutoff. In capillary sampling 2, four venous samples with corresponding capillary samples were above the clinical cutoff and 1/4 (25%) was predicted below the cutoff. Prediction of venous sample from capillary sampling 1 showed a difference in prediction error of 0.064–0.053 = 0.011 μg/l compared to the results of Åstrand et al. [12]. Prediction of venous sample from capillary sampling 2 showed a difference in prediction error of 0.064–0.058 = 0.006 μg/l compared to Åstrand et al. [12].

## Discussion

The only comparative study of capillary and venous serum S100B protein level in adult patients with traumatic brain injury was performed by Åstrand et al., and the methodology of the present study was based on that study. However, some methodological differences between the study by Åstrand et al. and the present study exist. Åstrand et al. drew two comparative capillary samples only on the first day of patient inclusion. To improve this, we drew two capillary samples at each sampling event and allowed for several sampling events in each patient with IH [12].

All other diagnoses that might have resulted in elevated serum S100B protein level due to brain damage, increased permeability of the blood-brain barrier, and peripheral damage were excluded. There is no scientific evidence to support that including these diagnoses would bias sampling, but the rationale for exclusion was that if serum S100B protein level from other sources potentially could affect the venous/capillary distribution of serum S100B protein level, excluding these diagnoses would reduce bias in the present study.

The range of the venous serum S100B protein level in the study population matches that of previously published concentrations in mTBI. Age and gender matching between healthy volunteers and patients with intracranial hemorrhage was not performed as it was only the serum S100B protein levels that were relevant to this study and not the specific patient traits.

The median serum S100B protein level of 0.08 μg/l in patients with intracranial hemorrhage was close to the median value (0.07 μg/l) of patients without intracranial hemorrhage [5]. This can in part be explained by the fact that the patients with intracranial hemorrhage were not always sampled within 6 h of trauma and were sampled over several days which could have led to a normalization in serum S100B protein levels. The time that elapses from trauma to sampling event is important when using S100B as a rule-out blood test but should not affect correlation or agreement and is therefore not discussed further.

Even though the differences between samples in patients with and without IH illustrated in Fig. 2 were statistically significant and more capillary samples drawn from patients with IH were above the clinical cutoff than below (illustrated by the red line of Fig. 2), this is not enough to support using capillary sampled S100B. The correlation and agreement are more important. The correlation between capillary and venous samples was very low and the difference statistically significant (Fig. 5). This low correlation cannot be explained by the small amount of time that had elapsed between sampling of venous serum S100B protein and capillary serum S100B protein as they were sampled just minutes apart. The very steep slope of regression lines and haphazard scattering of samples in the Bland-Altman analyses were interpreted as a total lack of agreement (Fig. 6). Neither the correlation nor the agreement between capillary samples were interpreted as good enough for a rule-out blood test as this probably requires very little inter-sample variation in samples drawn just minutes apart that theoretically should be identical.

Most capillary/capillary samples were within the limits of agreement. The low median bias, small slope of regression line, and higher correlation coefficient of capillary versus capillary samples compared to capillary versus venous samples indicated a certain level of both correlation and agreement between capillary samples. However, the limits of agreement are very wide and not appropriate for a cutoff blood test, at least if the cutoff is derived from serum samples and not acquired from the testing of capillary samples' predictive ability (Fig. 4). The line of regression in venous/capillary sampling showed a steep upward slope indicating that the mean difference was not constant. It became larger in higher capillary concentrations of serum S100B protein level compared to venous samples (Fig. 6). A feasible explanation could be that the highest capillary values in this study were false high values and the low number of high venous values prevents us from making a good analysis of the agreement in high-range samples. The line of regression in capillary samples showed a trend towards less agreement in the higher range (Fig. 4). This indicated that the mean difference was not constant, but varied to a lesser degree compared to capillary versus venous samples.

When applying the prediction equation to capillary samples in the present study, the mean prediction error was smaller than in the previous study by Åstrand et al., but the 25% incorrect classification below the clinical cutoff makes this method inappropriate for ruling out IH. The correlation coefficient between capillary samples presented by Åstrand et al. is high, but judging from the figures included by Åstrand et al., it appears to be in the lower at serum S100B protein level concentrations around the clinical cutoff. This might explain the high percentage of erroneously classified as below clinical cutoff [12].

As put forth by Bouvier et al. (2016), the time from sampling to analysis might play a role in the level of S100B and this time was not been recorded in the current study [4]. To improve concordance between venous and capillary sampled protein S100B, it might be beneficial to record this time and to keep it as short as possible to avoid bias from lysed leukocytes.

Commonly, capillary analysis volume ranges from 20 to 50 μl. The present method for serum S100B protein level assay, which requires 400 μl of blood, rendered 13.5% samples that could not be analyzed due to insufficient blood volume. This makes capillary blood for the serum S100B protein level assay difficult to use in the clinical setting. Operators with more capillary sampling expertise may improve the possibility of acquiring more samples with accurate sampling volume. Regardless of operator expertise, however, capillary samples of this size may be contaminated with extracellular fluid extracted by fingertip manipulation and fluids from other cells torn at the site of skin perforation. Therefore, capillary S100B analysis should not be attempted until it can be performed on smaller volumes of blood.

A weakness of the present study is that it is assumed that two consecutive venous samples would be identical. If this is not the case, we have no way of detecting this error. There are no previous studies that investigate this subject.

Larger molecules such as serum S100B protein might be difficult to analyze accurately in capillary blood. Troponin I is of similar molecular weight, but no scientific research investigating capillary analysis has been published. Capillary cortisol (0.362 kDa) shows a good correlation with venous samples, and this method requires very small sample volumes. It is difficult to know if it is the small molecule, small sample volume, or both that makes this analysis successful [23]. Capillary analysis of white blood cells, differential or platelet count, and hemoglobin (which are all large molecules) can differ significantly from venous analysis [24]. Although this might be acceptable in the clinical setting when analyzing continuous variables, it is not for a test with a fixed cutoff as the basis for clinical decision making.

Based on the present study, we cannot recommend using capillary serum S100B protein sampled and analyzed in accordance with the methodology of the present study in the setting of mTB. This would require much narrower limits of agreement and better correlation and agreement between venous serum S100B protein levels and capillary serum S100B protein levels over the entire spectrum of serum S100B protein concentrations commonly found in patients with mTBI. The wide limits of agreement and poor correlation and agreement between venous serum S100B protein levels and capillary serum S100B protein levels do not preclude using capillary serum S100B protein level as a means to rule out IH. However, the correlation and agreement between capillary samples presented in the present study make it unlikely that capillary serum S100B protein samples can be used to rule out IH. It is possible that repeated, averaged capillary samples would be more representative of the venous value and reduce the sampling bias. This in combination with an assay that would allow smaller sampling volumes and shorter time from sampling to analysis might be a way to design a future study of the predictive ability of capillary protein. Particular focus on time from trauma to sampling, several consecutive capillary samples to further evaluate consistency between capillary samples and receiver operator curve analysis of different capillary serum S100B protein levels as a cutoff to rule out intracranial hemorrhage would be beneficial. For correlation plots and logarithmized correlation plots of venous and averaged capillary values, please see Additional file 2: Figure S1 and Additional file 3: Figure S2.

## Conclusions

The results of this study indicate that correlation and agreement between capillary and venous samples are low, and because of this, we cannot recommend studies on capillary serum S100B protein level to rule out intracranial hemorrhage in mTBI. The prediction equation was not accurate enough in predicting venous protein S100B level from capillary protein S100B level.

Given the limitations of the current sampling and analysis methods of capillary protein S100B protein level, we conclude that evaluating its predictive ability to rule out intracranial hemorrhage should be withheld until more reliable methods can be incorporated into the study design.

## Additional files

Additional file 1: **Table S1.** Dispersion of serum protein S100B levels in patients with intracranial hemorrhage and healthy volunteers. (DOCX 14 kb)

Additional file 2: **Figure S1.** Correlation plot Venous and Average Capillary S100B. (PNG 62 kb)

Additional file 3: **Figure S2.** Log Correlation plot Venous and Average Capillary S100B. (PNG 73 kb)

## Data Availability

Data are available from the corresponding author upon reasonable request.

## Abbreviations

CT: Computerized tomography
IH: Intracranial hemorrhage
IQR: Interquartile range
mTBI: Mild traumatic brain injury
*R*^2^: Determination coefficient
S100B: Calcium-binding protein beta of the S100 family
SNC: Scandinavian Neurotrauma Committee
SPSS: Statistical Package for the Social Sciences
TBI: Traumatic brain injury
