# Supplementary material for: Prospective comparison of capillary and venous brain biomarker S100B: capillary samples have large inter-sample variation and poor correlation with venous samples
Source: Int J Emerg Med. 2019 Sep 2;12:26. doi: 10.1186/s12245-019-0239-6 (PMC6719369; doi:10.1186/s12245-019-0239-6)
Supplement: Supplementary file 1 — Table S1. Dispersion of serum protein S100B levels in patients with intracranial hemorrhage and healthy volunteers. (DOCX 14 kb) [file 12245_2019_239_MOESM1_ESM.docx]

|  |  | Serum protein S100B level | | |
| --- | --- | --- | --- | --- |
| Patient category | Sampling | Median | IQR (mg/l) | Median difference/(IQR (mg/l) |
| Intracranial hemorrhage group and healthy volunteers | Capillary 1 | 0.12 | 0.075-0.21 | -0.01/-0.43-0.03 |
|  | Capillary 2 | 0.13 | 0.08-0.22 |  |
| Intracranial hemorrhage group | Capillary 1 | 0.18 | 0.11-0.29 | -0.01/-0.05-0.07 |
|  | Capillary 2 | 0.17 | 0.11-0.29 |  |
| Healthy volunteers | Capillary 1 | 0.1 | 0.07-0.14 | -0.01/-0.05-0.02 |
|  | Capillary 2 | 0.09 | 0.06-0.17 |  |

Heading

Dispersion of serum protein S100B levels in patients with intracranial hemorrhage and healthy volunteers.

Additional file 1: Table S1. IQR – Interquartile Range
