# Supplementary figures and images for: Prospective comparison of capillary and venous brain biomarker S100B: capillary samples have large inter-sample variation and poor correlation with venous samples
Source: Int J Emerg Med. 2019 Sep 2;12:26. doi: 10.1186/s12245-019-0239-6 (PMC6719369; doi:10.1186/s12245-019-0239-6)

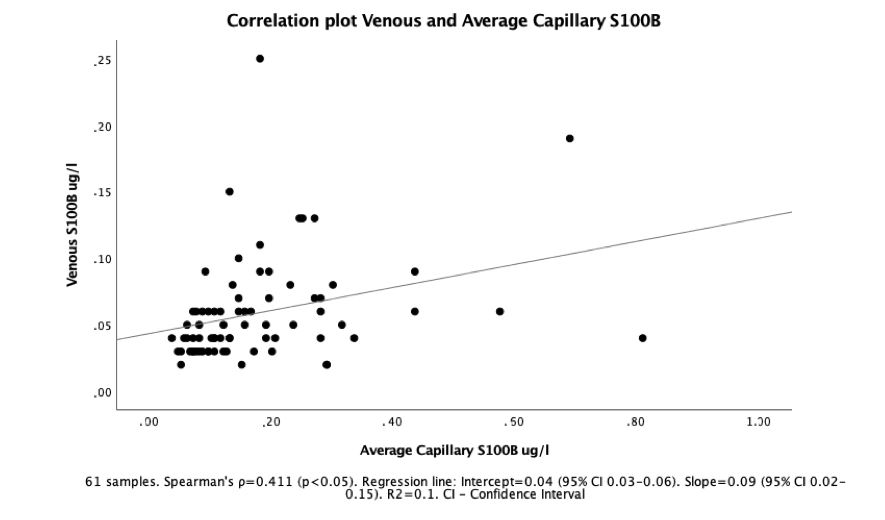

Supplement: Supplementary file 2 — Figure S1. Correlation plot Venous and Average Capillary S100B. (PNG 62 kb) [file 12245_2019_239_MOESM2_ESM.png]

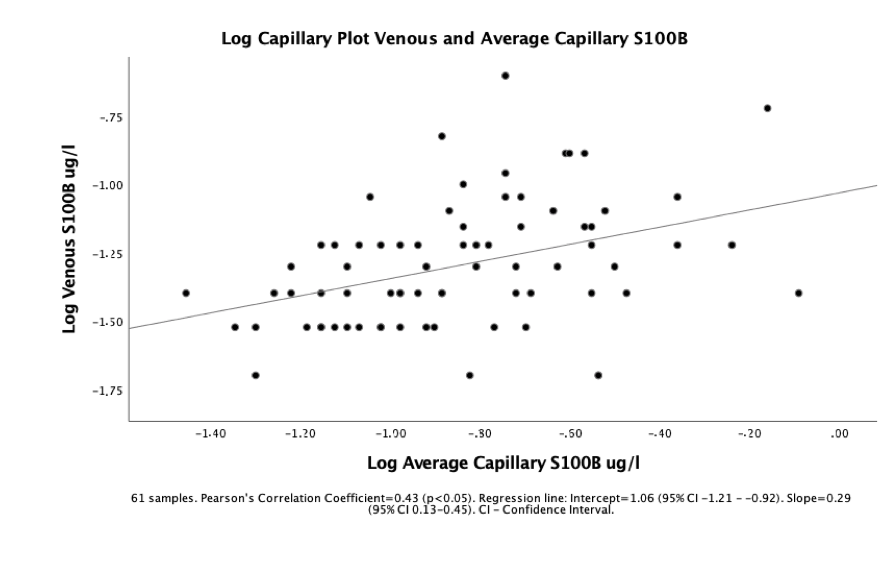

Supplement: Supplementary file 3 — Figure S2. Log Correlation plot Venous and Average Capillary S100B. (PNG 73 kb) [file 12245_2019_239_MOESM3_ESM.png]
